# The Improved Prognosis of Hypoplastic Left Heart: A Population-Based Register Study of 343 Cases in England and Wales

**DOI:** 10.3389/fped.2021.635776

**Published:** 2021-07-06

**Authors:** Kate E. Best, Nicola Miller, Elizabeth Draper, David Tucker, Karen Luyt, Judith Rankin

**Affiliations:** ^1^Population Health Sciences Institute, Newcastle University, Newcastle upon Tyne, United Kingdom; ^2^Leeds Institute of Health Sciences, University of Leeds, Leeds, United Kingdom; ^3^Public Health England National Congenital Anomaly and Rare Disease Registration Service, London, United Kingdom; ^4^Department of Health Sciences, University of Leicester, Leicester, United Kingdom; ^5^Congenital Anomaly Register and Information Service, Swansea, United Kingdom; ^6^Bristol Medical School, University of Bristol, Bristol, United Kingdom

**Keywords:** congenital heart, hypoplastic left heart, survival, epidemiology, register, congenital anomalies

## Abstract

**Background:** Hypoplastic Left Heart Syndrome (HLHS) is a severe congenital heart defect (CHD) characterised by the underdevelopment of the left side of the heart with varying levels of hypoplasia of the left atrium, mitral valve, left ventricle, aortic valve and aortic arch. In the UK, age 12 survival for cases born between 1991 and 1993 was 21%. UK survival estimates corresponding to cases born between 2000 and 2015 were improved at 56%, but survival was examined up to age five only. Contemporary long-term survival estimates play a crucial role in counselling parents following diagnosis. The aim of this study was to report survival estimates up to age 15 for children born with HLHS or hypoplastic left ventricle with additional CHD in England and Wales between 1998 and 2012.

**Methods:** Cases of HLHS notified to four congenital anomaly registers in England and Wales during 1998–2012, matched to Office for National Statistics mortality information, were included. Kaplan-Meier survival estimates to age 15 were reported. Cox regression models were fitted to examine risk factors for mortality.

**Results:** There were 244 cases of HLHS and 99 cases of hypoplastic left ventricle co-occurring with other CHD, with traced survival status. Kaplan-Meier survival estimates for HLHS were 84.4% at age 1 week, 76.2% at 1 month, 63.5% at age 1 year, 58.6% at age 5 years, 54.6% at age 10 years, and 32.6% to age 15 years. The Kaplan-Meier survival estimates for cases of hypoplastic left ventricle co-occurring with additional CHD were 90.9% at age 1 week, 84.9% at 1 month, 73.7% at age 1 year, 67.7% to age 5 years, 59.2% to age 10 years, and 40.3% to age 15 years. Preterm birth (*p* = 0.007), low birth weight (*p* = 0.005), and female sex (*p* = 0.01) were associated with mortality.

**Conclusions:** We have shown that prognosis associated with HLHS in the twenty first century exceeds that of many previous population-based studies, likely due to improvements in intensive care technologies and advances in surgical techniques over the last few decades.

## Introduction

Hypoplastic Left Heart Syndrome (HLHS) is a severe congenital heart defect (CHD) characterised by the underdevelopment of the left side of the heart with varying levels of hypoplasia of the left atrium, mitral valve, left ventricle, aortic valve and aortic arch (1). Survival was not possible until the 1980s when the Norwood procedure was introduced (2, 3). Since then, improvements in antenatal screening, preoperative management, resuscitation, and surgical techniques such as right ventricle-to-pulmonary artery conduits and the hybrid procedure for neonates too unstable (e.g., very preterm or with other comorbidities) to undergo the Norwood procedure, have further improved the prognosis for HLHS patients (4, 5). However, the impact of right ventricle-to-pulmonary artery conduits and the hybrid procedure on longer term prognosis remains under debate (6). While not the standard approach, survival in children and adults with HLHS may also have improved due to increased frequency and improved survival associated with heart transplants (7).

Today the standard treatment for individuals with HLHS is to undergo the Norwood stage I procedure (reconstruction of the left track and modified Blalock Taussig shunt or Sano technique) within the first 2 weeks of life, the Norwood stage II procedure (Hemi fontan, Glenn, or partial cavo-pulmonary bypass) procedure between 4 and 6 months of age and finally the Norwood stage III (Fontan final phase, full cavo-pulmonary bypass) procedure between 18 months and 4 years of age (8). According to the UK's National CHD audit, 30 day post-operative survival for the Norwood, Glenn and Fontan procedures exceeds 90% (9). However, when they occur with a hypoplastic left ventricle, other forms of CHD such as atrioventricular septal defect (AVSD), common arterial trunk (CAT), and double outlet right ventricle (DORV) may also be surgically treated with the Fontan procedure, but not necessarily after a Norwood stage I surgery as is the case for HLHS (10, 11). These forms of CHD with a hypoplastic left ventricle, often known as functional univentricular hearts, have better prognosis than HLHS and so the post-surgical survival rates for HLHS specifically is likely to be lower (12). A recent UK study reported 5 year survival for cases of HLHS born between 2000 and 2015 was 56.3%, although this study did not ascertain those who did not undergo any interventional treatment (13). Outside of the UK, recent age five survival rates range between 18.8% in New Zealand to 72% in Denmark (14, 15). Longer-term estimates of survival from birth are important for clinicians counselling parents when HLHS is diagnosed prenatally, as they may aid decision making regarding continuation of pregnancy. Given that individuals with palliated CHD will require follow-up throughout their lives, contemporary survival estimates are also important for commissioners whose role is to ensure services are in place for the growing population of adults with CHD.

The primary aim of this study was to report contemporary survival estimates up to age 15 for children born with HLHS or hypoplastic left ventricle in addition to other CHD subtypes in England and Wales, and to examine risk factors for mortality among these cases.

## Materials and Methods

### Data

**Figure 1:**
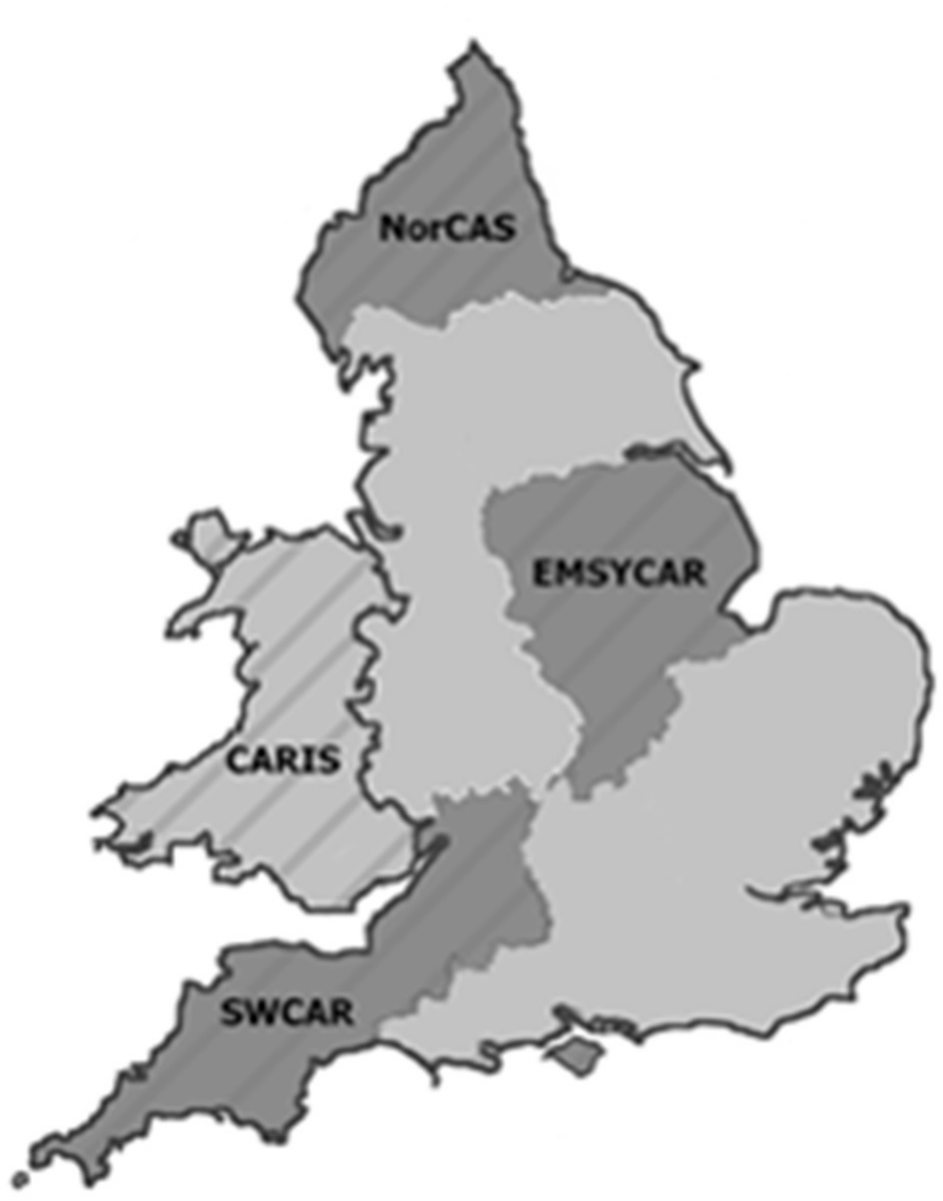
Map showing the area covered by the congenital anomaly registers.

The British and Irish Network of Congenital Anomaly Research Database (BINOCARD) was a collaborative network of regional population-based congenital anomaly registers (16), which was superseded in England by a national system, the National Congenital Anomaly and Rare Diseases Registration Service (NCARDRS) in 2015 (17). Each regional register prospectively collected data on congenital anomalies occurring in the pregnancies of women residing in their geographically defined populations (Figure 1). Data were obtained from three registers in England and the register for Wales, covering ~28% of the birth population of England and Wales. The Northern Congenital Abnormality Survey (NorCAS), established in 1985, covered 32,000 births per year in the North East of England and North Cumbria; the East Midlands and South Yorkshire Congenital Anomaly Register (EMSYCAR), established in 1997 covered 74,000 births per year; the South West Congenital Anomaly Register (SWCAR), established in 2002, covered 50,000 births per year in South West England; and the Congenital Anomaly Register and Information Service (CARIS), established in 1998, covers 35,000 births per year in Wales. Each register recorded up to eight congenital anomalies for each case. Anomalies were coded using the WHO International Classification of Disease (ICD) version ten, consistent with the European Surveillance of Congenital Anomalies (EUROCAT) guidelines (18). To ensure a high case ascertainment, congenital anomalies were notified to each register from a variety of sources including prenatal ultrasound departments, foetal medicine units, cytogenetic laboratories, regional cardiology centres, pathology departments, and paediatric surgery departments. CHD diagnoses were confirmed at surgery, by echocardiography or cardiac catheterisation, or at post-mortem.

The register data were linked to death registrations via NHS Digital in December 2017, using the “fuzzy matching” of infant name, sex, date of birth, and postcode at delivery. These identifier variables were removed or pseudo-anonymised (e.g., date of birth was replaced with year of birth only) following the linkage before the data were accessed by the researcher (KEB). While NHS Digital were able to provide the exact age of cases resulting in mortality, it was not possible to provide the exact age at case censorship for survivors as this would have allowed date of birth to be identified by the researcher (due to the linkage date being known). Therefore, age at censorship was estimated by subtracting the number of days between the midpoint of year of birth and December 2017. For each case with an English postcode at birth, NHS Digital assigned the English Index of Multiple Deprivation (IMD) score and rank. IMD is a measure of area-level socioeconomic deprivation calculated from income, employment, health, education, access to services, social environment, housing stress, living environment and crime (19, 20). IMD 2004, 2007, 2010 were used according to the year of birth of the case. It was not possible for NHS Digital to provide the Welsh IMD and so analysis of IMD was restricted to cases notified to the English registers only (80.8% of cases).

England and Wales population survival estimates at ages 1 month and 1 year were taken from the Office for National Statistics' online vital statistics for comparison with cases of HLHS and hypoplastic left ventricle with additional CHD (21).

### Case Inclusion

Cases coded as hypoplastic left heart (ICD 10: Q234) that were live born during 1st January 1998–31st December 2012 were included. The EUROCAT uses the same ICD 10 code (Q234) to describe both HLHS and severe hypoplastic left ventricle co-occurring with other CHD subtypes such as AVSD, CAT, and DORV (18). Given that these two forms of CHD are likely to have different survival rates, they were classed as two separate groups. HLHS was defined as cases coded with ICD Q234 but no other CHD codes except for those that commonly occur as part of HLHS: aortic valve stenosis/ atresia, mitral valve atresia/ stenosis, mitral regurgitation, aortic regurgitation, patent ductus arteriosus, atrial septal defect, and coarctation of aorta. We defined the remainder of cases as hypoplastic left ventricle with additional CHD, i.e., those cases coded as ICD Q234 in addition to any other CHD subtypes (Q20–26), excluding those listed above. Cases that occurred with extra-cardiac anomalies (ECA's, i.e., chromosomal anomalies or structural congenital anomalies of another organ system) or that occurred with additional CHD subtypes, were included. Twenty-eight (7.5%) cases were untraced, and these were excluded following descriptive statistics.

### Ethical Approval

The BINOCARD has approval from the Confidentiality Advisory Group of the Health Research Authority (PIAG 2-08I/2012), to hold data without consent and ethics committee approval (09/H0405/48) to undertake studies involving their data. The data linkage aspect of this study was given a favourable ethical opinion by the Newcastle & North Tyneside 2 Local Research Ethics Committee (13/NE/0188) and the Confidentiality Advisory Group of the Health Research Authority [5-08(b) 2013].

### Statistical Analysis

The associations between untraced survival status and demographic (categorical) variables were examined using χ^2^ tests of association or Fisher's exact tests. Kaplan-Meier survival estimates were estimated at ages 1 week, 1 month, 1, 5, 10, and 15 years and according to period of birth (categorised as 1998–2005 and 2006–2012). Survival estimates were not reported (numerically or graphically) where the number of cases at risk was <5, given that this can produce unstable estimates.

Hazard ratios (HRs) were estimated using univariable Cox regression models for the following predictors: extra-cardiac anomalies [none (i.e., isolated CHD), structural (e.g., digestive system congenital anomalies), chromosomal (e.g., Down syndrome)], maternal age at delivery (<20, 20–34, ≥35 years), birth weight (low: <2,500 g, average: 2,500–3,999 g, high: ≥4,000 g), gestational age at birth (preterm: <37 weeks, term: 37–41 weeks, post-term ≥42 weeks), infant sex (male, female) and IMD at birth [quintile 1 (most deprived), quintile 2, quintile 3 quintile 4, quintile 5 (least deprived)], plurality (singleton or multiple).

The proportional hazards assumption was checked using log-log plots, Schoenfield residulas, and by comparing hazard ratios for different categorisations of survival time. *P* < 0.05 was considered statistically significant and analyses were performed in Stata 14.

## Results

There were 371 live born cases included in the study, giving a live birth prevalence of 1.5 (1.4, 1.7) per 10,000 live births. Survival status was traced for 343 (92.5%) cases. Untraced cases were more common in cases born earlier in the study period (*p* < 0.001) and in cases born with a low or high birth weight (*p* = 0.002). The proportion of traced cases also varied by register (*p* = 0.001), with 82.5% of CARIS cases traced up to 100% of SWCAR cases. There was no association between traced status and gestational age at delivery (*p* = 0.17), maternal age at delivery (*p* = 0.35), infant sex (*p* = 0.90), plurality (*p* = 0.14), ECAs (*p* = 0.45), or IMD (*P* = 0.48). The 28 untraced cases were excluded from further analysis.

### Case Characteristics

There were 244 cases defined as HLHS and 99 defined as hypoplastic left ventricle with additional CHD. Cases of HLHS were most commonly: isolated CHD (*n* = 220, 90.2%), born at >37 weeks gestation (*n* = 195, 83.7% of cases with available gestational age), with average birth weight (*n* = 171, 71.8%), to mothers aged 20-34 (*n* = 160, 69.0%), of male sex (*n* = 140, 57.3%) and to mothers residing in the most deprived areas (IMD quintile 5: *n* = 35, 30.2%) (Table 1).

**Table 1:** Birth characteristics of cases of HLHS and hypoplastic left ventricle with additional CHD.

**Risk factors**	**All cases *n* (% of 343)**	**HLHS *n* (% of 244)**	**Hypoplastic left ventricle with additional CHD *n* (% of 99)**
**Register**			
CARIS	66 (19.2)	57 (23.4)	9 (9.1)
EMSYCAR	162 (47.2)	103 (42.2)	59 (59.6)
NorCAS	50 (14.6)	41 (16.8)	9 (9.1)
SWCAR	65 (19)	43 (17.6)	22 (22.2)
**Year of birth**			
1998–2005	137 (39.9)	109 (44.7)	28 (28.3)
2006–2012	234 (68.2)	157 (64.3)	77 (77.8)
**Gestational age**			
Preterm (<37 weeks)	50 (14.6)	38 (15.6)	12 (12.1)
Term/Post-term (≥37 weeks)	280 (81.6)	195 (79.9)	85 (85.9)
Missing	13 (3.8)	11 (4.5)	2 (2.0)
**Birth weight**			
Low (<2,500 g)	63 (18.4)	41 (16.8)	22 (22.2)
Average (2,500–3,999 g)	240 (70)	171 (70.1)	69 (69.7)
High (≥4000g)	32 (9.3)	26 (10.7)	6 (6.1)
Missing			
**Maternal age**			
<20	44 (12.8)	29 (11.9)	15 (15.2)
20–34	225 (65.6)	160 (65.6)	65 (65.7)
≥35	61 (17.8)	43 (17.6)	18 (18.2)
Missing	44 (12.8)	29 (11.9)	15 (15.2)
**Sex**			
Female	139 (40.5)	104 (42.6)	35 (35.4)
Male	204 (59.5)	140 (57.4)	64 (64.6)
**Socioeconomic deprivation (IMD)**			
Least deprived Q1	38 (11.1)	27 (11.1)	11 (11.1)
Q2	38 (11.1)	25 (10.2)	13 (13.1)
Q3	43 (12.5)	21 (8.6)	22 (22.2)
Q4	61 (17.8)	46 (18.9)	15 (15.2)
Most deprived Q5	92 (26.8)	64 (26.2)	28 (28.3)
Missing	71 (20.7)	61 (25.0)	10 (10.1)
**Extra cardiac anomalies**			
Isolated CHD	297 (86.6)	220 (90.2)	77 (77.8)
Structural ECA	27 (7.9)	12 (4.9)	15 (15.2)
Chromosomal ECA	19 (5.5)	12 (4.9)	7 (7.1)

*HLHS, Hypoplastic Left Heart Syndrome; ECA, Extra Cardiac Anomaly, IMD, Index of Multiple Deprivation*.

Cases of hypoplastic left ventricle co-occurring with additional CHD, were more commonly: isolated CHD (*n* = 77, 77.8%), born at >37 weeks gestation (*n* = 85, 87.6%), of average birth weight (*n* = 69, 71.1%), to mothers aged 20–34 (*n* = 65, 66.3%), of male sex (*n* = 64, 64.6%) and to mothers residing in the most deprived areas (IMD quintile 5: *n* = 28, 31.4%) (Table 1). These cases most commonly occurred with ventricular septal defect (*n* = 29), AVSD (*n* = 17), DORV (*n* = 13), interrupted aortic arch (*n* = 10), and transposition of the great vessels (*n* = 8), with 41 cases having “other” or “unspecified” CHD.

### Survival and Trends in Survival

**Figure 2:**
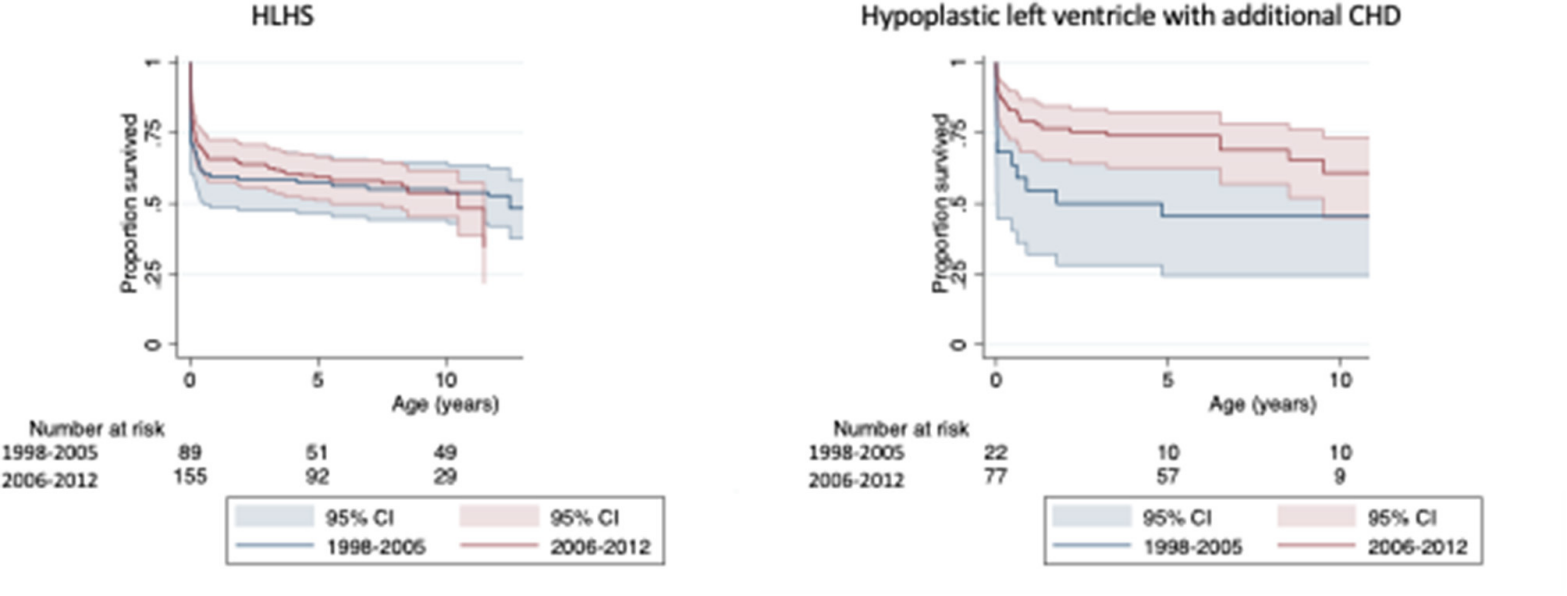
Kaplan-Meier survival curves by year of birth.

Kaplan-Meier survival estimates for cases of HLHS born between 1998 and 2012 are shown in Table 2, with 84.4% of cases surviving the first week of life, 76.2% the first month, 63.5% to age 1 year, 58.6% to age 5 years, 54.6% to age 10 years, and 32.6% to age 15 years. Compared to cases born 1998–2005, survival of cases born 2006–2012 appeared to be slightly increased up to age 5 years (Figure 2), but similar thereafter. Due to violation of the proportional hazards assumption, survival in the two era's was not formally compared.

**Table 2:** Kaplan-Meier survival estimates for HLHS and hypoplastic left ventricle with additional CHD born between 1998 and 2012.

**Age**	**All cases Survival (95% CI)**	**HLHS Survival % (95% CI)**	**Hypoplastic left ventricle with additional CHD Survival % (95% CI)**
1 week	86.3 (82.2, 89.5)	84.4 (79.2, 88.4)	90.9 (83.3, 95.2)
1 month	78.7 (74, 82.7)	76.2 (70.4, 81.1)	84.9 (76.1, 90.6)
1 year	66.5 (61.2, 71.2)	63.5 (57.2, 69.2)	73.7 (63.9, 81.3)
5 years	61.2 (55.9, 66.2)	58.6 (52.2, 64.5)	67.7 (57.5, 75.9)
10 years	56.2 (50.6, 61.4)	54.6 (48.1, 60.7)	59.2 (47.5, 69.2)
15 years	34.4 (25.8, 43.1)	32.6 (23.5, 42.0)	40.3 (19.6, 60.2)

*HLHS, Hypoplastic Left Heart Syndrome*.

The Kaplan-Meier survival estimates for cases of hypoplastic left ventricle with additional CHD were slightly higher compared to cases of HLHS, with 90.9% of cases surviving the first week of life, 84.9% the first month, 73.7% to age 1 year, 67.7% to age 5 years, 59.2% to age 10 years, and 40.3% to age 15 years. However, the difference in survival between the two phenotypes was not statistically significant (log-rank test: *p* = 0.14). The association between year of birth and mortality among cases of hypoplastic left ventricle with additional CHD did not reach statistical significance (log-rank test: *p* = 0.09), survival appeared to be increased in those born 1998–2005 vs. 2006–2012 (Figure 2).

The survival rates at ages 1 month and 1 year for cases of HLHS and hypoplastic left heart variant were significantly lower than those of the general population of England and Wales (99.5 and 99.7%, respectively, test of proportions: *p* < 0.001).

### Predictors of Survival

In all cases combined, there were significant associations between mortality and gestational age at delivery (*p* = 0.007), birth weight (*p* = 0.005), and infant sex (*p* = 0.01) (Table 3). One year survival estimates increased with increasing gestational age at delivery (52.0, 67.5, and 91.7% for preterm, term and post-term births), although the association was only statistically significant in preterm vs. term births (HR = 1.77, 95% CI: 1.21, 2.57). One year survival estimates increased with increasing birth weight (57.1, 66.7, and 81.3% for low, average, and high birth weight), with the association being statistically significant in low vs. average birth weight cases (HR = 1.51, 95% CI: 1.09, 2.23) and high vs. average birth weight (HR = 0.50, 95% CI: 0.25, 0.98). Males had decreased risk of mortality compared to females (HR = 0.67, 95% CI: 0.50, 0.91). There was no evidence of an association between mortality and plurality (*p* = 0.82), maternal age (*p* = 0.17), socioeconomic deprivation (*p* = 0.65) or ECAs (*p* = 0.86).

**Table 3:** Risk factors for mortality in cases of HLHS or hypoplastic left ventricle with additional CHD born 1998–2012.

**Risk factors**	***N* (%)**	**Age 1 year Kaplan-Meier survival estimate**	**Hazard ratio (95% CI)**	***P*-value**
**Gestational age**				0.007
Preterm (<37 weeks)	50 (14.5)	52.0 (37.4, 64.7)	1.77 (1.21, 2.57)	
Term (37–41 weeks)	270 (78.3)	67.5 (61.6 (72.8)	1 (reference category)	
Post-term (≥42 weeks)	12 (3.5)	91.7 (53.9, 98.8)	0.72 (0.29, 1.76)	
Missing	13 (3.8)			
**Birth weight**				0.005
Low (<2,500 g)	63 (18.3)	57.1 (44.0, 68.3)	1.51 (1.09, 2.23)	
Average (2,500–3,999 g)	240 (70.1)	66.7 (60.3, 72.2)	1 (reference category)	
High (≥4,000 g)	32 (9.3)	81.3 (63.0, 91.1)	0.50 (0.25, 0.98)	
Missing	8 (2.3)			
**Maternal age**				0.17
<20	44 (13.0)	54.6 (8.8, 67.8)	1.42 (0.92, 2.19)	
20–34	225 (65.5)	68.9 (62.4, 74.5)	1 (reference category)	
≥35	61 (17.7)	63.9 (50.6, 74.6)	1.31 (0.88, 1.93)	
Missing	13 (3.8)			
**Sex**				
Female	139 (40.3)	59.7 (51.1, 67.3)	1 (reference category)	0.01
Male	204 (59.7)	71.1 (64.3, 76.8)	0.67 (0.50, 0.91)	
**Socioeconomic deprivation (IMD)**				0.65
Least deprived Q1	38 (11.0)	73.7 (56.6, 94.9)	1 (reference category)	
Q2	38 (11.3)	55.3 (38.3, 69.3)	1.57 (82.9, 2.97)	
Q3	43 (12.5)	69.8 (53.7, 81.2)	1.06 (54.9, 2.04)	
Q4	61 (17.7)	67.2 (53.9, 77.5)	1.18 (0.65, 2.17)	
Most deprived Q5	93 (27.0)	67.4 (56.8, 76.0)	1.20 (0.68, 2.12)	
Missing	71 (20.6)			
**Plurality**				0.82
Singleton	320 (93.3)	66.3 (60.8, 71.1)	1 (reference category)	
Multiple	15 (4.3)	66.7 (37.5, 84.6)	1.09 (0.51, 2.33)	
Missing	8 (2.0)			
**Extra-cardiac anomalies**				0.86
Isolated	297 (86.7)	67.3 (61.7, 72.4)	1 (reference category)	
Structural ECA	27 (7.9)	55.6 (35.2, 71.8)	1.25 (0.72, 2.17)	
Chromosomal ECA	19 (5.5)	68.4 (42.8, 84.4)	1.23 (0.67, 2.27)	

*HLHS, Hypoplastic Left Heart Syndrome, ECA, Extra-Cardiac Anomaly, IMD, Index of Multiple Deprivation*.

## Discussion

### Summary

Based on this population-based study of 343 traced cases of HLHS or hypoplastic left ventricle with additional CHD born in England and Wales, we have shown that survival to age 5 years in the twenty first century is as high as 59 and 68%, respectively. Survival was slightly greater amongst cases with hypoplastic left ventricle with major CHD compared to HLHS, although the difference was not statistically significant. This study shows that the greatest risk of mortality in those with HLHS or hypoplastic left ventricle and additional CHD occurs during the first week of life, although survival continued to decrease over the 15 years of follow-up. Survival estimates were increased in the later cohort (2006–2012 vs. 1998–2005) although for cases of HLHS they may only have been increased until age 5 years.

### Strengths

The main strength of this study is the use of high-quality data derived from four regional population-based congenital anomaly registers. The registers were notified of cases from multiple sources including prenatal ultrasound, cardiology databases, cytogenetics and post-mortems. As a result, there is likely very high ascertainment of cases. The ascertainment of cases at birth as opposed to at surgical intervention is a further strength as this has enabled pre-operative mortalities to be captured. However, this also means that we have included in our estimates those that opted for palliative care, which will have deflated the survival estimates. A recent survey of European surgeons reported that parental intention to treat was 95% in the UK, with the remaining 5% opting for comfort care (22). According to a US study of military personnel, the proportion of parents opting for comfort care is minimal (3%) since the 2000's and so these cases probably make up a very small proportion of our cohort (23).

A further strength of this study is that we attempted to separate cases of HLHS and cases of hypoplastic left ventricle with additional CHD subtypes such as DORV or AVSD. We have shown that survival estimates may vary between these two types of cases, with cases of hypoplastic left heart occurring with additional CHD having slightly improved prognosis when compared to classic cases of HLHS. Combining these cases may also over inflate the survival estimate associated with classic HLHS. Ideally, cases of HLHS would be coded as those that underwent a Norwood stage I procedure only, but unfortunately the data we used was not linked to information on surgical intervention. As a result we may have misclassified some cases of CHD with hypoplastic left ventricle as HLHS. Additionally, we may have misclassified cases that occurred with ventricular septal defects as we were not able to identify the specific phenotype. Where muscular VSD could occur as part of HLHS, the same cannot be said for conotruncal VSD. However, misclassification likely accounted for only minority of cases of VSD. However, our approach allows cases with HLHS that did not survive until Norwood stage I to be included in the analysis.

### Limitations

This study has some limitations. Firstly, “fuzzy matching” was used to link the congenital anomaly register data to death registrations. As a result, we were not able to trace the survival status of 28 cases. This could have caused bias in our survival estimates if the survival statuses of these cases were significantly different to the cases that were traced. These cases were slightly more likely to be of low or high birth weight compared to the traced cohort and were more likely to have been born earlier in the study period, which suggests they might have had poorer survival than the traced cases. Given that 93% of the cohort were traced, the impact of any biases caused by excluding the untraced cases will be minimal. Future linkage studies will benefit by linking using National Health Service (NHS) number, which is now routinely recorded by the NCARDRS. An additional weakness was that our analysis of socioeconomic status (IMD) was restricted to cases born to mothers resident in England only, which reduced the power of this analysis. Moreover, due to the low prevalence of hypoplastic left ventricle with additional CHD, we may not have had adequate power to detect statistically significant differences in survival between the two time periods and when comparing to HLHS survival.

The BINOCARD did not routinely link to hospital episode statistics, so we were not able to present pre- or post-operative survival estimates relating to cardiac surgeries. Post-operative survival estimates for cases treated in the UK are available via NICOR, with 30 day post-operative survival for the Norwood, Glenn and Fontan procedures exceeding 90% (9). There is very limited information on modern pre-operative mortality rates, with the most recent UK study of cases born between 1992 and 1993 reporting that 32% of cases died with no intervention (12). However, it is not clear what proportion of these had an intention to treat. Moreover, we could not attribute mortalities to heart transplants which may have occurred at various points of the life course and likely represent post-neonatal deaths. While paediatric heart transplants are rare in the UK in general (*n* = 23 in 2012, according to the UK National Transplant Registry), the proportion of heart transplants for CHD doubled in the UK between 2002 and 2012 (24). The proportion of HLHS cases that result in transplant in the UK have not been recently reported, but according to the Children's Heart Federation are likely to account for a very small number of cases due to the availability of donor hearts within that age range (25). Linkage between the data recorded by the congenital anomaly registers and hospital episode statistics is now theoretically possible via the NCARDRS and is currently being explored by the authors. The estimates in this study could be used in conjunction with surgical survival rates now provided by the National CHD audit to build a more accurate picture of HLHS prognosis (9).

We found improved survival among cases of hypoplastic left ventricle with additional CHD compared to HLHS. However, this group represents a variety of different phenotypes which may have variable prognoses. Further research into the specific combinations of CHD subtypes is required to provide more specific survival estimates for these cases.

The BINOCARD recorded routinely collected demographic information for each case, but did not hold clinical information on treatments or interventions. Due to the small number of available demographic risk factors, the lack of clinical predictors and the relative rarity of some of the characteristics (e.g., post-term birth and high birthweight), we did not attempt to perform a multivariable analyses or examine causality. Mediation analyses could be performed to examine whether the negative impact of low birth weight is accounted for primarily by the effects of preterm birth. In future studies, a path analysis may be applied to examine the complexity of the association between socioeconomic deprivation and mortality, although we found little evidence of an association in the univariable setting.

### Comparison to Previous Studies

A previous UK study reported age 12 survival to be 21% for cases born during 1992–1993 (12), which is consistent with cases born in the early part of our study period. More recently, 5 year survival for cases of HLHS born in 2000–2015 was 56.3% in the UK, which is comparable to the 5 year survival reported in our study (58.6%) (13). According to the UK National CHD audit of surgeries performed between 2015 and 2016, 30 day post-operative survival was 92.8% following the Norwood procedure, 98.2% following Glenn procedure and 99.6% following Fontan (9). This suggests the greatest risk of mortality is associated with the stage 1 repair, which usually occurs in the first 1 or 2 weeks of life (8). This is reflected in our study which shows the highest decrease in survival during the first week and between the first week and first month of life. However, this high hazard rate may also represent cases that died pre-operatively or those that opted not to treat.

The survival estimates provided in this study relate to children born in England and Wales, who were entitled to free health care provided by the NHS. Our survival estimates may not be representative of other populations with different health care systems, for example in the USA where the health care system is not publicly funded, and CHD outcomes vary according to health insurance status (26). Perhaps for this reason, our survival estimates exceed those of 60 cases born in Atlanta (USA) between 1999 and 2005 (27), who had 10 year survival of 42.5% compared to our estimates of 54.7% (HLHS) and 48.0% hypoplastic left ventricle with additional CHD between 1998 and 2005. Differences in survival may also be due to differences in infant transplant provision, which is relatively uncommon in the UK but may remain as a parental choice in some centres in the USA (28). Conversely, in Denmark, which has a publicly funded health care system, 10 year survival for 143 children born with univentricular hearts between 2003 and 2015 exceeded our survival estimates at 72%, although cases were ascertained based on surgical procedures so some pre-operative mortalities may not have been ascertained (29). A different regional study in Denmark that ascertained 308 cases from a CHD register reported much lower 5 year survival estimate of 18.8% for cases of univentricular heart born between 2000 and 2009 (14). These differences in survival estimates may also be related to the definition of HLHS and the accuracy of coding, the decision to terminate following a prenatal diagnosis or to opt for comfort care, which vary by country and centre (22). In New Zealand, 5 year survival for cases of HLHS born between 2006 and 2010 was 62.1% although this estimate is based on 29 cases ascertained on surgical procedures (15). The applicability of our survival estimates to cases born in developing countries, where there is a paucity of population-based research into HLHS survival, is also not clear.

We found that preterm birth and low birth weight were associated with an increased risk of mortality, which is consistent with previous research (14, 27, 30). These factors may influence the age at which surgeries are performed, the stability at the time of operation, lung development and the technical aspects of surgical reconstruction, which can negatively impact on survival (31). Similar to previous studies, we also found no evidence that ECAs increase the risk of mortality (27). While this is not the case for most other CHD subtypes (32), HLHS is perhaps so clinically severe that additional congenital anomalies are overpowered in terms of mortality. In cohorts from Denmark and the USA, increased mortality rates among those with high vs. low levels of neighbourhood poverty has been reported (14, 27, 30). We did not find any statistically significant evidence of an association with IMD, which could perhaps be reflective of the differences in our health care systems. We found no association between mortality and plurality or maternal age with is consistent with several previous studies, although none of these are based on UK populations (14, 27, 30).

In our study, female sex was associated with an increased risk of mortality, possibly because these cases were more likely to be HLHS as opposed to hypoplastic left ventricle with additional CHD. Three population-based studies did not report a significant association between infant sex and mortality (14, 27, 30), although in two the mortality rate among females was greater (14, 27). In a USA based study, surgical mortality was significantly greater in females following the Norwood procedure, but not following the Glenn or Fontan procedures (33). Another US study also reported an increased risk of mortality in females following the Norwood procedure, although this was of borderline statistical significance (15.5% in males vs. 30.0% in females, *p* = 0.07) (34). Speculatively, females with HLHS could have increased likelihood of being born with a more severe HLHS phenotype, which would increase the gender disparity in mortality, but there is no research to corroborate this currently.

### Implications

We have shown survival up to age 15 for those born with HLHS has improved compared to previous studies based on earlier cohorts. This information is important for counselling parents when their child is diagnosed with HLHS. However, the survival estimates reported in this study need to be considered alongside data on quality of life and morbidity. There is some evidence that children with HLHS experience lower quality of life, increased risk of neurodevelopmental delay, behavioural problems and decreased functional status (35, 36). Given that survival was very rare until the 1990s and 2000s, there is also a paucity of information known on longer term survival and quality of life in adults living with palliated HLHS.

## Data Availability Statement

Data was obtained from the National Congenital Anomaly and Rare Diseases Registration Service and linked to ONS death registrations via NHS Digital. For NCARDRS data requests please email phe.ncardrs@nhs.net. Linked data requests will require additional ethical approvals.

## Ethics Statement

The studies involving human participants were reviewed and approved by Newcastle & North Tyneside 2 Local Research Ethics Committee (13/NE/0188). Written informed consent from the participants' legal guardian/next of kin was not required to participate in this study in accordance with the national legislation and the institutional requirements.

## Author Contributions

KB was responsible for the statistical analysis of the data and drafting the initial manuscript. NM, ED, KL, and DT provided the data and critically reviewed and commented on the manuscript. JR, along with KB, conceived and designed the study and had input into the drafting and critical appraisal of the manuscript. All authors contributed to the article and approved the submitted version.

## Conflict of Interest

The authors declare that the research was conducted in the absence of any commercial or financial relationships that could be construed as a potential conflict of interest. The handling Editor declared a past co-authorship with the authors KB, JR.
